# Role of ACTN4 in Tumorigenesis, Metastasis, and EMT

**DOI:** 10.3390/cells8111427

**Published:** 2019-11-13

**Authors:** Dmitri Tentler, Ekaterina Lomert, Ksenia Novitskaya, Nikolai A. Barlev

**Affiliations:** 1 Institute of Cytology, Russian Academy of Sciences, 4 Tikhoretsky ave., 194064 Saint Petersburg, Russia; e.lomert@gmail.com (E.L.); asianov@mail.ru (K.N.); nick.a.barlev@gmail.com (N.A.B.); 2 Moscow Institute of Physics and Technology, Dolgoprudny, 141701 Moscow, Russia

**Keywords:** ACTN4, tumorigenesis, metastasis, epithelial-mesenchymal transition, cell motility, cancer cell proliferation

## Abstract

The actin-binding protein ACTN4 belongs to a family of actin-binding proteins and is a non-muscle alpha-actinin that has long been associated with cancer development. Numerous clinical studies showed that changes in ACTN4 gene expression are correlated with aggressiveness, invasion, and metastasis in certain tumors. Amplification of the 19q chromosomal region where the gene is located has also been reported. Experimental manipulations with ACTN4 expression further confirmed its involvement in cell proliferation, motility, and epithelial-mesenchymal transition (EMT). However, both clinical and experimental data suggest that the effects of ACTN4 up- or down-regulation may vary a lot between different types of tumors. Functional studies demonstrated its engagement in a number of cytoplasmic and nuclear processes, ranging from cytoskeleton reorganization to regulation of different signaling pathways. Such a variety of functions may be the reason behind cell type and cell line specific responses. Herein, we will review research progress and controversies regarding the prognostic and functional significance of ACTN4 for tumorigenesis.

## 1. Introduction

The ACTN4 protein belongs to the family of actin binding proteins. It was initially described as a non-muscle α-actinin strongly associated with the motility of cancer cells [1]. Since then, a number of different functions have been assigned to this protein, ranging from the cytoplasmic structural organization of the cytoskeleton [1,2] to the regulation of nuclear transcription factor activity [3,4,5,6,7] and to the replication of viruses [8]. Phenotypic manifestations of certain ACTN4 gene mutations include the malformation and dysfunction of kidneys [9]. While the molecular mechanisms responsible for this phenotype are not well understood and require further investigation, it is accepted that mechanistically ACTN4 affects cell cycle and the migration of cells [10], which indicates its importance in tumor development and metastasis. Consequently, it is therefore not surprising that ACTN4 has now become a focus of various biomedical studies. While the expression of ACTN4 was shown to be elevated in certain types of tumors, suggesting its role as an oncogene [11,12,13], in other cases it has been shown to exhibit tumor suppressor activity [10,14]. Apparently, ACTN4 may play opposing roles in cancer development and progression and its effect is strictly cell type and tissue specific. Consequently, such opposing findings can create difficulties in the extrapolation of published data to other types of well-characterized cancer models, and herein we review the evidence and mechanisms for ACTN4 activity and its regulation proposed to date.

## 2. ACTN4 as a Predictive Marker for Cancer Development

Since its original description as a protein associated with cancer invasion [1] many attempts were made to correlate ACTN4 expression with cancer prognosis and the success of particular treatment regimes.

For example, the ACTN4 gene copy number was found to be a reliable predictive factor for salivary gland carcinoma and tongue cancer [15,16]. FISH-detected gene amplifications correlated with a shorter overall survival time. Surprisingly, this correlation has not been observed for high ACTN4 expression levels when determined by IHC. Similarly, in a study of oral squamous cell carcinoma samples, there was no statistically significant correlation between the ACTN4 expression and patients’ survival rates as judged by IHC, although ACTN4 was expressed at higher levels in the invasive areas [17].

The data of independent clinical studies suggests that approximately only 30–40% of high-expressing ACTN4 tumors estimated by IHC also display increased gene copy numbers detected by FISH [11,18]. Thus, the gene amplification may not be the only reason behind an increased ACTN4 protein level. Hence, considering both ACTN4 gene dosage and its expression makes them more reliable prediction factors for various aspects of tumor development.

In line with this, the ACTN4 gene copy number determination using FISH has been adapted as a predictive marker for the efficiency of chemotherapy in cases of locally advanced pancreatic cancer [19,20]. Patients with the normal copy number showed a better outcome following chemo and radiotherapy treatment, whereas the high copy number patients showed diminished responses to the therapy. Amplification of ACTN4 was also found to correlate with the survival outcomes of lung adenocarcinoma patients [21]. The gene dosage was determined by FISH to tissue microarrays that contained 543 patient samples, out of which 15% displayed ACTN4 amplification. The amplification frequency was significantly higher in samples with advanced (II–IV) stages and poor histological differentiation. These data correspond to a microarray expression study of resected non-small cell lung tumors that identified the ACTN4 gene as the only strong predictor of overall survival, as it was highly expressed in a poor survival group [22].

Analysis of patient information from the public database showed significant benefits of adjuvant chemotherapy against non-small cell lung cancers (NSCLC) expressing ACTN4 mRNA [23]. However, ACTN4 mRNA levels in NSCLC cell lines showed no effects on conventional anticancer drug resistance. Taking into account the experimental data, the authors proposed that the therapy might impair metastatic activity of cancer cells with high ACTN4 expression. This notion was further supported by the following IHC study of lung adenocarcinoma patients after resection of the primary tumor [24]. The results also highlighted the possibility that the patients may benefit from therapy only when tumors exhibit high ACTN4 expression levels. This further supported the data that high ACTN4 levels were involved in the NSCLC recurrence through tumor metastases. Statistically significant correlation between ACTN4 staining and lymph node metastasis for NSCLC was obtained by IHC analysis of tissue microarray [25]. Comparative RNA-sequencing analysis of samples from the primary tumor, local lymph nodes and brain metastases from a single lung adenocarcinoma patient [26] revealed high expression levels of ACTN4 in brain metastatic tissue but not in the lymph nodes, primary tumor, or normal lung tissues. Similarly, an expression microarray study identified ACTN4 and DUSP6 as two-gene prognostic indicators of a high risk of recurrence for patients with lung squamous cell carcinoma (SCC) following surgery [27].

Notably, a specific splice variant of ACTN4 mRNA with an alternative exon 8 was detected in small-cell lung cancer (SCLC) [28]. The encoded protein was different from the ubiquitous one in three amino acids, was localized in the nucleus, and was not associated with F-actin structures. The splicing variant was strongly associated with shorter survival times of patients lacking distant metastases [29]. In another study, this variant of ACTN4 protein was predominantly observed in high grade neuroendocrine lung tumors, including 60% of SCLC and 51% of large cell neuroendocrine carcinomas (LCNEC) [30]. The frequency of relapse after surgery was higher in the cases positively stained for the variant protein, and it was a significant indicator of negative prognosis. These data suggest that the ACTN4 variant is somehow related to tumor metastatic development, although the nature of this relationship is not yet clear.

A proteomic search for protein markers associated with estrogen receptor (ER) status, tumor grade and lymph node status in a set of breast cancer samples [31] revealed an association between the ACTN4 protein and the lymph node status in high grade tumors. Surprisingly, no association with ER status was observed, although ACTN4 has been described as an ER-co-activator in several experimental studies [5,32,33]. These results were later confirmed by qRT-PCR analysis of ACTN4 mRNA in blood serum of breast cancer patients [34], where high ACTN4 expression was associated with the high tumor grade and lymph node metastasis but was independent of ER, PR (progesterone receptor), or HER2 status. Another recent study showed that the mRNA level of ACTN4 was significantly elevated in breast cancers of the metastatic phenotype, and in triple-negative breast cancer tumors, lacking ER, PR, and HER2 [35].

Broad analysis of the clinical data suggests that the copy number and expression level of the ACTN4 gene seem to be independent and mutually exclusive predictive markers. In general, amplification of the ACTN4 gene is associated with a poor prognosis [12,16,18,21], a high histological grade (less differentiated tumors) [15,21], and a lower efficiency of therapy [12,18,19,20]. However, the correlation between the ACTN4 gene dosage and different cancer characteristics varies between studies. Besides, these differences may well result from variations in the amplified region. According to a CGH analysis [36], this chromosomal region may include other genes, which can also be responsible for inducing oncogenic effects. In this instance, ACTN4 amplification appears to have greater predictive value than changes of its expression level [12,15,19].

The predictive value of high ACTN4 expression does have a tendency to vary significantly between different types of tumors (Table 1). Here, one of the most commonly associated traits is lymph node metastasis, found in patients with pancreatic [11,37], lung [25], colorectal [38], esophageal [39] and breast [31,34] tumors. The association of metastasis to distal organs has been identified for ovarian [40], pancreatic [11,37], and lung [26] cancers. Invasion into the surrounding tissues correlates with high expression in bladder cancer [41,42], infiltrating gliomas [43] and thyroid cancer [44]. Therefore, there is a profound association between high ACTN4 expression and the migratory potential of tumor cells, although the data does vary between different studies of the same type of cancer.

Post-translational modifications are also known to regulate proteolysis of ACTN4 [45]. Investigation of splice isoforms, key post-translational modifications and factors, and into regulating ACTN4 expression, may reveal novel prognostic markers associated with certain cancer phenotypes.

## 3. ACTN4 and EMT-Related Tumorigenic Activity of Cancer Cells

Generally, ACTN4 over-expression induces invasive phenotypes in most of the cancer cell lines studied, whereas ACTN4 knockdown decreases this pathophysiological manifestation. Anecdotally, the initial studies on the involvement of ACTN4 in the tumor growth showed that the over-expression of ACTN4 exhibited tumor suppressive activity in cancer cells [10,14]. However, the following extensive evidence supporting the oncogenic potential of ACTN4 has been collected, and it is now firmly accepted that the over-expression of ACTN4 is associated with tumorigenesis in most types of cancer.

### 3.1. EMT Program in Cancer

The phenomenon of epithelial-to-mesenchymal transition (EMT) is widely attributed not only to a cell transformation process during the embryonic development, but also to a loss of epithelial features by cancer cells of the epithelial origin. EMT is deemed important for the migration of epithelial cancer cells from the primary tumor to form metastases in distant organs (for review, see references [50,51]). The process may be induced by a number of signaling pathways, including TGFβ, EGF, HGF, Notch, FGF, Wnt, IGF, and other ligands that activate the expression of the key EMT-inducing transcription factor families: Snail, Twist, and Zeb (EMT-TFs) [52,53]. Importantly, EMT is considered to be a stepwise process, which includes various intermediate phenotypes often referred to as partial EMT [50,53,54]. These hybrid partial EMT phenotypes are maintained in balance between EMT-TFs and pro-epithelial microRNAs (miR-34 and miR-200), which form negative feedback loops [53].

Mechanistically, to undergo EMT, epithelial cells must lose contacts with neighboring cells, reorganize cytoskeletons, change their shape from globular to spindle-like and acquire migration abilities. All these events seem to be reversible and are controlled by specific, yet overlapping, pathways [55].

Changes in cadherin/catenin adhesion complexes play a central role in disrupting cell-cell contacts [56]. Thus, one of the key markers of EMT is a decrease in the expression of E-cadherin and its replacement with mesenchymal cadherins (e.g., N-cadherin and R-cadherin). This process is referred to as the cadherin switch. Down-regulation of the CDH1 gene encoding E-cadherin occurs via binding of EMT-TFs to CDH1 promoter. The exact choice of the repressive factor seems to be cell-type specific and may depend on the epigenetic chromatin modifications [57,58]. Loss of E-cadherin leads to a release of β-catenin into the cytoplasm where it is either degraded by a GSK3β complex or, if stabilized by Wnt signaling, migrates into the nucleus and modulates the expression of a large number of genes [56]. In addition to E-cadherin loss, early EMT is signified by Snail-dependent repression of genes encoding occluding and claudins, which results in degradation of the tight junctions [59].

As mentioned before, realization of the EMT program in cancer cells is often incomplete. The cells with partial EMT may express both epithelial and mesenchymal markers depending on how far the program has advanced. In addition to mesenchymal markers, EMT cells are characterized by an increased expression of fibronectin and matrix metalloproteinases (MMPs), which promotes their migration abilities [52].

Recent investigations established a strong link between the partial EMT and cancer stem-like cells (CSCs) [53,54,60]. Investigations have demonstrated that EMT-TF are often enriched in cancers, including CSCs, and contribute to the acquisition of stem-like traits [61]. This finding proposes a novel explanation for the involvement of EMT in metastases via maintaining a population of circulating stem-like cells. Once deployed at a new niche, the cells may undergo a process which is the opposite of EMT: mesenchymal-epithelial transition (MET); and give rise to a new epithelial-like tumor.

Another related aspect covers the EMT-driven resistance of cancer cells to cytotoxic agents. Indeed, drug resistance is one of the CSC traits, which, for example, includes drug efflux by cell membrane transporter proteins like MDR1. In addition, some of the EMT-inducing pathways, like TGFβ and Wnt/β-catenin, are also associated with an increased resistance to anti-cancer treatment [60,62].

### 3.2. Functional Association of ACTN4 with EMT Factors

ACTN4 was demonstrated to influence different aspects of EMT progression listed above. Indeed, changes in cellular morphology from epithelial to mesenchymal induced by elevated ACTN4 expression have been described for both cancerous and non-cancerous cells. Increased motility and loosing epithelial features induced by the high expression of ACTN4 were also observed in NSCLC cells CL1-0 [25] and colorectal cancer cells DLD-1 [38,63]. ACTN4 over-expression in Madin-Darby Canine Kidney (MDCK) cells and several cervical cancer cell lines resulted in EMT-like changes, which included increased motility and invasiveness [64]. These changes were manifested by an increased expression of EMT markers SNAIL, vimentin and N-cadherin, and a decreased expression of E-cadherin. Moreover, the inhibition of Akt signaling suppressed the ACTN4-induced expression of SNAIL. Hence, the authors hypothesized that ACTN4 regulated EMT in cervical cancer cells by activating Akt signaling pathway whereas the augmentation of their proliferation was achieved by stabilization of the β-catenin protein.

### 3.3. ACTN4 and β-Catenin

A functional association of ACTN4 with β-catenin has been confirmed by several proteomic studies on different cell lines [35,63]. Using the co-IP/mass-spectrometry approach, ACTN4 was described as a protein partner of β-catenin in colorectal cancer cells upon the loss of E-cadherin [63]. In such cells, ACTN4 co-localized with β-catenin into actin-rich structures. This event was accompanied by cytoskeletal reorganization and enhanced cell migration. Similarly, a study showed the interaction of β-catenin with ACTN4 in breast CSCs. There, ACTN4 regulated the stability of β-catenin by inhibiting its proteasome degradation in an Akt/GSK3β-dependent manner [35]. Importantly, the ACTN4/β-catenin axis was shown to promote CSC traits in breast cancer cell lines. Expectedly, ACTN4 knockdown had the opposite effect.

Another ACTN4 protein interacting partner, NHERF1, targeted E-cadherin/β-catenin complexes at the cell membrane [65]. Over-expression of NHERF1 reduced the ACTN4 protein level by promoting its ubiquitination and proteasomal degradation, leading to actin cytoskeleton disassembly [66]. In accordance with this finding, NHERF1 knockdown augmented the ACTN4 protein level in cervical cancer cells [67]. Furthermore, the authors have shown that NHERF1 inhibits ACTN4-mediated stabilization of β-catenin, activation Wnt/β-catenin signaling, and hence suppresses the proliferation of cervical cancer cells. Collectively, ACTN4 seems to play an important role in the regulation of E-cadherin/β-catenin complexes and in the defining the β-catenin fate following E-cadherin loss in early EMT.

### 3.4. ACTN4 and NF-kappaB

NF-kappaB signaling is well known to be essential for EMT activation and progression. Activation of NF-kappaB induces EMT in Ras-transformed human mammary epithelial cells [68]. Significance of the RelA/p65 activation for TGFβ-triggered EMT has also been demonstrated in small airway epithelial cells [69]. Furthermore, the study has shown that RelA/p65 is situated up-stream of Wnt, JUN, and SNAIL signaling pathways. Functional RelA/p65 binding sites were found in the promoter regions of critical EMT-TFs genes: SLUG (SNAI2), TWIST1 and ZEB2 (SIP1) [70]. Recent findings suggested that ACTN4 affected the activity of NF-κB, thereby increasing the migration of cancer cells [71]. These authors revealed that the decrease of ACTN4 expression by specific shRNA attenuated the level of phosphorylation of the RelA/p65 subunit of NF-kappaB in osteosarcoma cells. This resulted in suppressed proliferation, migration, and metastasis. Reciprocally, the knockdown of RelA/p65 produced a similar inhibitory effect. Although osteosarcoma cells already have mesenchymal features, EMT-TFs are known to be involved in osteosarcoma pathogenesis (for review see e.g., reference [72]). Thus, ACTN4-dependent regulation of NF-kappaB activity may be an important factor responsible for their EMT status.

To substantiate the functional importance of this link, several papers have described the interaction of ACTN4 with RelA/p65 in the nucleus, which resulted in the co-activation of NF-kappaB-mediated transcription [6,7,73]. ACTN4 over-expression enhanced the negative effect of NF-kappaB activation on the proliferation of H1299 NSCLC cells [74]. However, there is evidence suggesting that in melanoma cells cytoplasmic NF-kappaB is activated by ACTN4 indirectly via its physical interaction with cIAP1 and RIPK1 [75]. Interestingly, the authors found that NF-kappaB and ACTN4 form a positive feed-forward loop whereby RelA transcriptionally up-regulates the ACTN4 gene, whose product in turn further activates NF- κB, resulting in enhanced survival, proliferation, and anchorage-independent growth of cancer cells.

Considering the data that ACTN4 is associated with chromatin remodeling proteins such as HDAC7 [5] and the INO80 complex [76], it is possible that ACTN4 plays an important role in modulating chromatin organization on the promoters of NF-kappaB target genes. Further investigations will shed light on the significance of the ACTN4/NF-kappaB axis in EMT.

### 3.5. ACTN4 Involvement in MDR

Since EMT reportedly confers multiple drug resistance to cancer cells, it is important to note that the over-expression of ACTN4 was noticed to parallel the accretion of drug resistance in several cancer cell lines. For example, the elevated level of ACTN4 was detected in cisplatin-resistant SK-N-AS cells, which exhibited enhanced migratory potential [77]. Likewise, ACTN4-expressing MCF-7 cells of the epithelial origin along with gaining radio-resistance also displayed the augmented expression of several mesenchymal markers including vinculin, integrin β1, and fibronectin. These features along with loss of E-cadherin strongly suggested that radio-resistance was associated with EMT [78]. Knockdown of ACTN4 by specific shRNA in these cells abolished all these morphological alterations and ultimately diminished their radio-resistance.

Several studies reported that the inhibition of Akt signaling affected cell motility and their drug resistance [78]. In this respect, the physical interaction of ACTN4 with Akt1 was described in several independent studies [79,80]. For example, ACTN4 co-localized with activated p-Akt in the membrane ruffles [78], and the attenuation of ACTN4 by siRNA prevented Akt phosphorylation and translocation to the cell membrane [79]. Since ACTN4/Akt-induced EMT may cause the resistance of cancer cells to DNA-damaging drugs [81,82], it is tempting to speculate that this mechanism is responsible for the failure of genotoxic anti-cancer therapies (Table 1).

More clues of possible involvement of ACTN4 in drug resistance come from the analysis of its interacting protein partners. For example, one of the ACTN4-binding proteins is the organic cation transporter protein SLC22A3, which is critical for the transport of several drugs through the membrane. Over-expression of SLC22A3 is associated with a high risk of incidence of prostate and colon cancers. Importantly, loss of this interaction facilitated cell invasion and formation of filopodia in normal esophageal cells [83]. In contrast, interaction with ACTN4 of the MDM2 binding protein (MTBP) abrogated ACTN4-mediated rearrangements of the actin cytoskeleton, thereby reducing the migratory activity of SaOs2-LM7 and U2OS osteosarcoma cells [2]. It should also be mentioned that the E3 ubiquitin ligase MDM2 is the major negative regulator of the tumor suppressor protein p53 [84]. Thus, an intriguing possibility emerges wherein ACTN4 is involved in the regulation of p53 stability and hence activation of apoptosis. The direct interaction of ACTN4 with DNaseY (DNAS1L3), which mediates DNA fragmentation during apoptosis, strengthens this hypothesis [85]. In line with this is another recent study that describes the involvement of ACTN4 in the p38-MAPK/p53 apoptosis pathway in human endothelial cells [86]. Collectively, it is plausible that ACTN4 may affect drug resistance by attenuating apoptosis in cancer cells.

### 3.6. ACTN4 Effects on Invasion and Migration of Cancer Cells

An important aspect of metastasis formation is the ability of cancer cells to migrate to other organs. In this respect, it is firmly established that the over-expression of ACTN4 promotes the migration of cells. ACTN4 has been reported to localize along stress fibers and to regulate the assembly and functioning of lamellipodia. This co-localization with structures involved in cell movement indicates its decisive role in the migration and invasion of cancer cells [87]. Importantly, analysis of ACTN4 distribution during the wound healing assay showed that it is up-regulated in cells along the wound edges and in cells migrating inside the wound [1,40].

Moreover, a number of extracellular signals, which cause reorganization of actin cytoskeleton, such as EGF, extracellular matrix proteins, and cytochalasin D also promote co-localization of ACTN4 with actin structures in epidermoid carcinoma cells A431 [73,88]. One of the groups demonstrated that PI3 kinase-mediated phosphorylation might be a key signal for the dissociation of α-actinin from integrins, which triggers the cytoskeletal remodeling [89]. Surprisingly, inhibition of PI3 kinase by wortmannin also causes the translocation of ACTN4 to the nucleus [1]. Curiously, nuclear translocation of ACTN4 was originally considered as an impairment of its cytoplasmic functions. However, subsequent studies have revealed its distinct, non-overlapping role in nuclear processes, like the regulation of transcription factor activity.

As discussed earlier, ACTN4-binding partners may determine its function in various processes, including cell adhesion. In this respect, ACTN4 interacts with the hemidesmosomal component, BP180 [90]. Furthermore, an interaction of ACTN4 with the cell adhesion molecule ICAM1 has been observed. Inhibition of this association resulted in decreased leukocyte extravasation, which is central to the innate immune response [91]. LIM kinase 1 (LIMK1), a key regulator of the cytoskeletal organization, was identified as an ACTN4 binding partner in SW480 colorectal cancer cells [92]. However, the exact functional significance of these interactions for EMT remains to be elucidated.

It has been demonstrated that changes in the ACTN4 gene expression level affect the formation of structures responsible for cancer cell motility. Astrocytoma cells form fewer lamellipodia after ACTN4-specific gene silencing by shRNA [48]. The study of special types of membrane protrusions called invadopodia showed that the knockdown of ACTN4 resulted in the disruption of these structures in cancer cells. Indeed, ACTN4 was shown to co-localize with invadopodia markers (ortactin, dynamin II, and phosphotyrosine) in cancer cell lines of various origin, such as MDA-MB-231, RPMI7951, DLD-1, and SCC61 [38,63,93]. However, some of the cell lines, e.g., MCF7 or T47, did not form invadopodia spontaneously, even upon increased ACTN4 expression, which indicates that high expression of ACTN4 alone is not sufficient for this process [37,93].

An augmented migratory potential was reported for a large number of ACTN4-over-expressing cells, including osteosarcoma, colorectal cancer, squamous carcinoma, breast cancer, lung cancer, and others [2,11,17,21,23,35,63,71,93,94]. Collectively, elevated levels of ACTN4 seem to be associated with the mobility of the sub-membrane structures, the disassembly of cellular contacts, and higher migration ability in most of the cell lines studied.

ACTN4 has also proven to be necessary for efficient amoeboid-type invasiveness of melanoma cells [95]. Loss of ACTN4 incapacitated WM1158 cells to restore their amoeboid morphology in 3D collagen I gel, which in turn led to an impaired invasive ability in 3D matrix. Surprisingly, elongated mesenchymal cells with ACTN4 knockdown migrated faster than control cells on a collagen I-coated surface. Similarly, knockdown of ACTN4 suppressed the invasiveness of gastric metastatic MKN 7 and NCI-N87 cell lines [47]. In contrast, cell-matrix adhesion was seen to be enhanced under these conditions. Over-expression in WM983b cells of either wild type ACTN4 or ACTN4Y11/13E mutant, which mimics EGF-stimulated ACTN4 phosphorylation at tyrosines 11 and 13, significantly altered cell morphology from mesenchymal to amoeboid-like [45]. The ACTN4Y11/13E expressing cells displayed more profound morphological changes, which also resulted in a higher invasive potential through matrigel but a lower migration speed across a collagen-coated surface. Thus, ACTN4 phosphorylation at tyrosines 11 and 13 increases the invasiveness but not the migration. Collectively, the data suggests that ACTN4 is involved in several modes of cellular movement, i.e., ensuring either cellular movement across a flat surface or invasiveness through dense matrix.

However, in several cell lines, changes in ACTN4 expression can affect both the invasive and migratory potential of cells. For example, the knockdown of ACTN4 expression in ovarian carcinoma cell line DOV13, non-small-cell lung carcinoma A549, bladder cancer cells T24, J82, and KU19-19, leads to a reduction in both invasiveness and migration [23,40,41,42].

Thus, alterations in ACTN4 expression levels are often manifested with changes in cell motility, migration and invasive potential. The effects, however, seem to be cell line-specific. Nevertheless, it can be assumed that ACTN4 influences cellular motility by reorganizing the motility apparatus, thereby inhibiting or potentiating metastasis. 

## 4. Conclusions and Prospects

Taken together, the clinical and experimental data presented herein clearly suggest that ACTN4 expression levels may alter the migration ability and invasive potential of different cancer cells. Often, outcomes are associated with a poor patient prognosis, although this is highly dependent on the cancer type. The results of experimental manipulations with ACTN4 expression show that the outcome may vary even between different cell lines of the same origin. Nevertheless, some generalizations can still be drawn from the data described above.

A source of controversy on the role of ACTN4 in cancer stems from the fact that most of the clinical investigations use the IHC approach to evaluate ACTN4 expression. Despite being convenient for clinical investigations, this method does not provide the most accurate comparison of protein levels in different samples. Furthermore, the mechanisms controlling transcription, mRNA splicing, and post-translational modifications of the ACTN4 gene have not really been explored. Splice variants of ACTN4 [4,32,96] may escape detection with routine IHC analysis, and there is still no information on why these rearrangements appear at all. Even less is known about factors that control the transcription of the ACTN4 gene except the very assumption that they may exist. Importantly, mutations in the ACTN4 promoter that correlate with decreased expression have been identified in patients with the focal segmental glomerulosclerosis [97].

**Figure 1:**
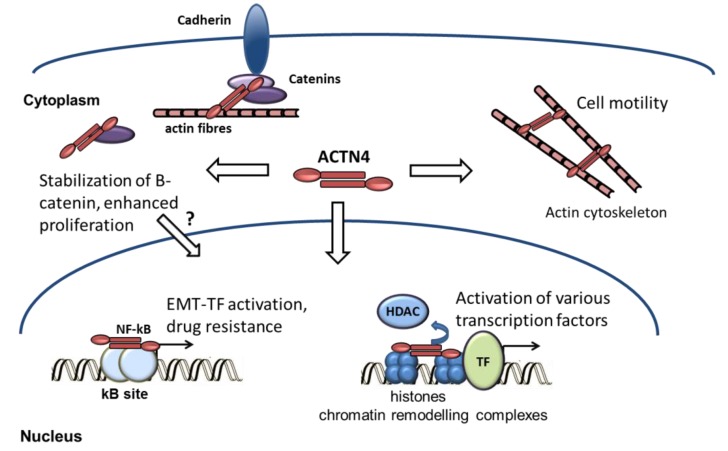
Involvement of ACTN4 in cellular processes. ACTN4 is involved in multiple signaling pathways and intracellular processes. The main function of cytoplasmic ACTN4 is to crosslink actin filaments, thus affecting the F-actin dynamics and migration ability of cancer cells [87]. ACTN4 also regulates intercellular contacts via the interaction with cadherin/catenin adherent junctions. Upon loss of E-cadherin, ACTN4 stabilizes β-catenin released from the junction complexes and hence inhibits its proteasomal degradation [35,64]. Evidently, ACTN4 migrates to the nucleus where it affects the Wnt/β-catenin pathway. However, the mutual translocation of ACTN4 and beta-catenin into the nucleus has not been described. Upon becoming nuclear, ACTN4 interacts with a large number of transcription-related proteins, influencing expression of various genes. This includes the association of ACTN4 with NF-kappaB promoting the co-activation of RelA-mediated transcription [6,7]. The latter, in turn, may increase drug resistance and activation of the EMT program. Alternatively, ACTN4 may enhance transcription via the association with chromatin remodeling complexes [5,76], thereby affecting various aspects of cancer development.

Another explanation to pleiotropic effects of ACTN4 relies on the notion that ACTN4 is involved in the regulation of different signaling pathways (Figure 1). To date, the close ACTN4 association with members of the Wnt/β-catenin, NF-kappaB and nuclear receptors signaling pathways have been established. Importantly, all these factors play pivotal roles in the process of EMT. The growing number of identified protein partners indicates that the list of pathways and processes ACTN4 is involved in may be extended further. Thus, the proteomic data demonstrates the interaction of ACTN4 with ribonuclear proteins of the hnRNP family [49,98], but the functional significance of this association remains elusive. In line with this, investigation of ACTN4 protein partners will facilitate the development of anti-cancer compounds that directly interfere with these tumor-promoting protein-protein interactions. For instance, ellagic acid disrupts the ACTN4 interaction with β-catenin, which subsequently leads to the suppression of breast cancer cell proliferation as well as ablating tumor growth and metastasis in an MMTV-PyMT breast cancer mouse model [35]. Another potential ACTN4-interacting drug called HAMLET (Human Alpha-lactalbumin Made LEthal to Tumor cells) was shown to trigger cancer cell detachment followed by their death in vitro and in vivo [99]. However, a serious consideration for pharmacological targeting ACTN4 requires an in-depth evaluation of its activity in each type of cancer cell. This is important because of the ACTN4 binding a high number of protein partners resulting in pleiotropic functions of this protein in human cells. It may well be that responses to the ACTN4 protein level and/or localization reflect specific cell type-associated pathways. In this case, investigations of ACTN4 interactions may become a valuable asset to predictive value by combining its use with other tumor-specific markers. Another source of concern is that ACTN4 deficiency may potentially be compensated by another member of the ACTN family, ACTN1 [100,101]. Despite that ACTN1 and ACTN4 were shown to form heterodimers in the yeast two-hybrid system [102], several studies demonstrated that these highly homologous proteins play distinct roles in the regulation of cell adhesion, motility, and signal transduction [48,93,103].

Taken together, this information highlights the importance of ACTN4 in the regulation of dissemination and proliferation of cancer cells. Further experiments are required to decipher the molecular mechanisms that ACTN4 engages to promote EMT, metastases, and proliferation of cancer cells as well as developing precise therapies that will target the oncogenic features of ACTN4 while sparing its functions in normal cells.

## Figures and Tables

**Table 1:** Association of ACTN4 expression with clinical features of cancers.

ACTN4 Status	Cancer Type	Detection Method	Association
**Gene amplification**	Fallopian tube carcinoma [36]	Comparative genomic hybridization analysis and multiplex ligation-dependent probe amplification	Stages II and III
	Ovarian cancer [12,18]	FISH, IHC	Chemoresistance and poor prognosis
	Locally advanced pancreatic cancer [19]	FISH	Chemoresistance and poor prognosis
	Salivary gland carcinoma [15]	FISH	High histological grade and vascular invasions
	Tongue cancer [16]	FISH	Poor survival
	Lung adenocarcinoma [21]	Tissue microarrays	Advanced stages and poor histological differentiation
**Increased expression**	Ovarian cancer [46]	IHC and tissue microarrays	Advanced stages and poor survival
	Invasive pancreatic ductal carcinoma [11]	IHC	Metastases in lymph nodes and distal organs
	Oral squamous cell carcinoma [17]	IHC	Invasion areas; no correlation with survival
	Non-small cell lung carcinoma (NSCLC) [21,22,25]	cDNA microarrays [22], IHC [21,25]	Poor survival [21,22] Lymph node metastasis [25]
	Lung squamous cell carcinoma [27]	Microarray study	High risk of disease recurrence
	Brain metastatic tissue [26]	RNA-seq	Distant organ metastases
	Colorectal cancer [38]	IHC	Lymph node metastasis
	Gastric cancer [47]	RT Profiler PCR Array for Human Cell Motility	Stage IV
	Bladder cancer [41,42]	Immunoblotting [41], IHC [41,42]	Invasive tumors, high histological grade
	Esophageal cancer [39]	Tissue microarrays and IHC	Advanced stages, lymph node metastasis
	Breast cancer [31]	Targeted and discovery proteomic analysis (SRM and iTRAQ)	Positive lymph node status especially in high grade tumors
	Thyroid cancer [44]	IHC	Infiltrating tumors
	Glioma [43,48]	Immunoblotting [43] IHC [48]	Advanced stages
**Reduced expression**	Prostate cancer [49]	IHC, immunoblotting	Low and high Gleason grades
**Specific spliced mRNA variant **	Small-cell lung cancer [28,29,30]	cDNA sequencing [28,29,30] IHC [28,29]	Associated with distant metastasis of small-cell lung cancer [28,29] Expressed in 55% of high-grade neurocrine tumors and associated with poorer overall survival [30]

## Abbreviations

FSGS	familial focal segmental glomerulosclerosis
EMT	epithelial-mesenchymal transition
EMT-TFs	epithelial-mesenchymal transition transcription factors
FISH	fluorescent in situ hybridization
IHC	Immunohistochemistry
NSCLC	non-small cell lung cancer
SCLC	small-cell lung cancer
SCC	squamous cell carcinoma
CGH	comparative genomic hybridization
